# Spatio-temporal parameters and intralimb coordination patterns describing hemiparetic locomotion at controlled speed

**DOI:** 10.1186/1743-0003-10-53

**Published:** 2013-06-12

**Authors:** Lucio A Rinaldi, Vito Monaco

**Affiliations:** 1 Dipartimento di Area Critica Medico Chirurgica, P.T., Laboratorio di Analisi del Movimento, Universitá degliStudi di Firenze, viale Pieraccini, 18, 50139, Firenze, I, Italy; 2 Istituto di BioRobotica, Scuola Superiore Sant’Anna, P.za Martiri della Libertà, 33, 56127, Pisa, I, Italy

**Keywords:** Hemiparetic gait, Fixed speeds, Spatio-temporal parameters, Intralimb coordination, Stroke

## Abstract

**Background:**

Comparison between healthy and hemiparetic gait is usually carried out while subjects walk overground at preferred speed. This generates bias due to the lack of uniformity across selected speeds because they reflect the great variability of the functional level of post-stroke patients. This study aimed at examining coordinative adaptations during walking in response to unilateral brain damage, while homologous participants walked at two fixed speeds.

**Methods:**

Five patients with left and five with right chronic hemiparesis, characterized by similar level of motor functioning, were enrolled. Ten non-disabled volunteers were recruited as matched control group. Spatio-temporal parameters, and intralimb thigh-leg and leg-foot coordination patterns were used to compare groups while walking on a treadmill at 0.4 and 0.6 m/s. The likelihood of Continuous Relative Phase patterns between healthy and hemiparetic subjects was evaluated by means of the root mean square of the difference and the cross correlation coefficient. The effects of the group (i.e., healthy vs. hemiparetics), side (i.e., affected vs.unaffected), and speed (e.g., slow vs. fast) were analyzed on all metrics using the Analysis of Variance.

**Results:**

Spatio-temporal parameters of all hemiparetic subjects did not significantly differ from those of healthy subjects nor showed any asymmetry between affected and unaffected limbs. Conversely, both thigh-leg and foot-leg coordination patterns appeared to account for pathology related modifications.

**Conclusion:**

Comparisons between hemiparetic and healthy gait should be carried out when all participants are asked to seek the same suitable dynamic equilibrium led by the same external (i.e., the speed) and internal (i.e., severity of the pathology) conditions. In this respect, biomechanical adaptations reflecting the pathology can be better highlighted by coordinative patterns of coupled segments within each limb than by the spatio-temporal parameters. Accordingly, a deep analysis of the intralimb coordination may be helpful for clinicians while designing therapeutic treatments.

## Background

Compensatory strategies adopted by post-stroke patients to increase stability and efficiency of locomotion have been widely described in literature by spatio-temporal parameters, kinematic, and kinetic measures referring to walking both overground [1-3] and on treadmill [4,5]. On the whole, the gait of post-stroke patients is mainly characterized by reduced speed, stride length, and cadence, decreased angular excursions at leg joints, increased energetic cost, and asymmetry in kinematic and kinetic variables [3]. Furthermore, a recent study highlighted that post-stroke patients walk with different patterns between treadmill and overground and, in particular, the treadmill enhances the asymmetry between affected and unaffected limbs, decreases the self-selected speed and the step length, and increases stance and double support percentages [6].

A cerebrovascular accident has been also shown to significantly modify the coordinative relationship of segments within (i.e., intralimb coordination) and between (i.e., interlimb coordination) lower limbs [7-9]. Specifically, previous authors have noticed that the intralimb coordinative patterns of chronic hemiparetics walking overground at their preferred speed can be characterized by asymmetry between unaffected and affected sides [7], and that the botulinum toxin injections into the rectus femoris of the paretic side can improve the thigh-leg coordination patterns of both limbs [8]. In addition, it was observed that the increase in speed positively influences the interlimb coordination of both arm and lower limb movements of acute patients undergoing intense rehabilitative treatments [9].

One of the common features of all mentioned studies [4,6-9] is that participants were asked to walk at their self selected speeds, that is – as acknowledged by some authors [3,4,10] – the walking speed was not uniform across subjects, basically due to the inherent variability related to the cerebrovascular accident (i.e., different clinical picture, rehabilitative training, neuromuscular adaptation across patients).

Actually, previous authors have already highlighted the importance of considering gait metrics in relation to the walking speed when attempting to classify gait abnormality [11,12]. This is because the velocity can be viewed as a control parameter of the dynamic system that, when scaled, involves changes of coordination even within the gait of walking alone [7,13]. Furthermore, in a previous study, Monaco and colleagues [14] demonstrated that the gait analysis, when carried out at controlled and comparable speed, can better pinpoint features between young and elderly healthy subjects than the comparison at self-selected pace.

Concerning post-stroke patients, it is well known that the self-selected speed reflects specific compensative strategies related to their own degree of impairment such that patients walking in a wide range of preferred velocities are characterized by very different functional deficit [15]. However, the speed, per se, affects in a significant fashion gait patterns inducing relevant modifications of the hemiparetic locomotion [12,15,16]. As matter of the fact, the inherent instability of the affected limb prompts the patients to an early shift of the weight on the unaffected one inducing an asymmetric duration of stance and swing phases [15,16]. In spite of this, when patients are asked to walk at faster speeds, i.e., pace comparable to healthy subjects, they show a bilateral increment of joint angular excursions and muscle activation [17], an improved symmetry in double and single support portions [15,17], and a significant modification of the coordination of pelvis and trunk rotations [12]. The speed-related improvements of the hemiparetic gait seem to be due to more appropriate timing of lower limb muscles, better suited movement coordination, and possibly facilitation of intralimb and interlimb energy transfers [17].

According to these results, it is possible to hypothesize that part of data variability leading previous authors to characterize motor performance of post-stroke patients may have been ascribed to the wide range of speeds rather than to the pathology itself. Specifically, metrics used to describe motor deficits of locomotion, may lack of a suitable sensitivity when different groups of homogenous subjects (e.g., similar age, anthropometric features, severity of the deficit) are observed while walking at the same pace.

This methodological study aimed at investigating the attitude of both spatio-temporal parameters and intralimb coordination patterns to capture some of the neuromuscular adaptations of the hemiparetic locomotion. This goal has been achieved by comparing gait patterns of affected and unaffected sides in post-stroke hemiparetic patients to those of healthy subjects while walking on a treadmill at the same speeds.

## Methods

### Participants

Subjects with a single cerebrovascular accident and consequent hemiparesis were enrolled for this study. In order to define a group of patients with homogeneous level of motor functioning, the inclusion criteria were: (1) first-time stroke reported in the admitting medical chart located in a single cerebral hemisphere and resulting in a sensorimotor disturbance of one side; (2) no evidence of hemianopsia; (3) no evidence of severe cognitive or language dysfunctions that would have interfered with the ability to understand instructions; (4) no evidence of neglect; (5) stroke occurred between 6 and 12 months before the experimental session; (6) Fugl-Meyer Motor Assessment scale score referring to the Lower Extremities [18] greater than 75% (FMA LE). According to the Bamford classification [19], all subjects were affected by unilateral partial anterior circulation stroke infarct. At the time of testing, all subjects had already carried out long-term standard physical therapy.

Non-disabled individuals (NDs) were also recruited to serve as gender, age, height and weight comparable controls for the hemiparetic subjects (HMs). Healthy subjects exhibited normal leg joint range of motion and muscle strength, did not show any apparent gait abnormality, and walked at the same speed as their matched hemiparetic participants.

The protocol was approved by Local Ethical Committee and all subjects signed the informed consent form before data collection.

### Procedure and technical apparatus

All participants walked on a treadmill at 0.4 m/s (low speed, LS) and 0.6 m/s (high speed, HS), in accordance with values reported as typical by Olney and Richards for stroke patients [3]. The two experimental sessions were performed in random sequence.

In order to allow subjects to accustom themselves, they started walking without handrails but were supported by two persons standing at the sides of the treadmill. When subjects said they were ready, they continued walking without any support for at least 3 min so that the warm-up period was in accordance with literature [4]. Subsequently, data were recorded during a 30-second time window.

The position of twenty reflective markers, placed in accordance with the Davis protocol [20], was acquired using a five camera ELITE_PLUS_ System (BTS, Milano, Italy) with a sample frequency of 100 Hz. Estimated maximum measurement error was 1.2 mm.

### Data collection

Ten consecutive and bilateral stride cycles were collected for each participant and each walking session. Raw data were low pass filtered using a fourth-order, zero lag, Butterworth filter with cut-off at 6 Hz [21]. Initial contact and toe off were estimated in accordance with previous authors [22]. Spatio-temporal (ST) parameters recorded for both sides were: stride time (s); cadence (steps/min); stride length (m); stance time (% of the gait cycle); duration of initial double stance (DS1; % of the gait cycle) and single limb support phases (SS; % of the gait cycle).

The Continuous Relative Phase (CRP), as described by previous authors [23,24], was adopted to evaluate changes in limb coordination in the sagittal plane. Briefly, thigh, leg, and foot angular orientations with respect to the horizontal axis were computed for each time frame using smoothed coordinates, and their related angular velocities were estimated by calculating the first derivative. Then, the angular orientation-angular velocity phase plane was constructed and normalized to a unit circle to correct for both amplitude and frequency differences between segments. Subsequently, phase angles were calculated as the four-quadrant arctangent of the ratio between normalized angular velocity and position for each point of time series. Finally, CRP measures between thigh and leg (CRP_TL_) and between leg and foot (CRP_LF_) were obtained by subtracting the distal from the proximal phase angle so that CRP measures were in a range from 0° to 180°. CRP patterns were normalized from 0 to 100% of the stride cycle by linear interpolation.

### Statistical analysis

The hypothesis of normal distribution of all datasets and the homogeneity of variance of all homologous datasets were assessed by, respectively, the Kolmogorov-Smirnov normality test and the Levene's test. The normality test showed that about the 4% of all samples did not fit the normality distribution (p<0.05) and the Levene's test showed that all homologous datasets were characterized by equal variance. Since the ANalysis Of VAriance (ANOVA) is relatively robust with respect to violations of the normality assumption and more sensitive to heterogeneous variances [25], the preliminary analysis of the data samples allowed to confidently use the ANOVA as the main statistical test in this study. In addition, to further verify that the outcome of the tests involving not Gaussian data samples were robust, the same hypothesis were tested by using the nonparametric Friedman test that confirmed the results of the ANOVA.

Then, with respect to the ST parameters:

a two-way ANOVA of Speed (HS vs. LS) and Side (affected vs. unaffected), with repeated measures on both factors, was performed on ST parameters related to the hemiparetic group;

a two-way ANOVA of Group (HM vs. ND) and Speed (HS vs.LS), with repeated measures on Speed, was performed on ST parameters once to compare the affected side of post-stroke patients to healthy subjects, and again to compare the unaffected side of post-stroke patients to healthy subjects.

In order to estimate changes of CRP patterns due to the stroke, CRP_TL_ and CRP_LF_ related to the HM group were compared to those of ND subjects using the Root-Mean-Square Difference (RMSD) and the cross-correlation coefficient (ρ), to respectively highlight discrepancies in amplitude and temporal evolution between coordination patterns of healthy and hemiparetic subjects. Specifically, for each patient, each side (i.e., affected and unaffected) and each experimental condition (i.e., HS and LS), RMSD and ρ were computed comparing CRP_TL_ and CRP_LF_ of that patient with those obtained after averaging all homologous CRP patterns across ND subjects.

Then, a two-way ANOVA of Speed (HS vs. LS) and Side (affected vs. unaffected), with repeated measures on both factors, was performed on ρ_TL_, RMSD_TL_, ρ_FL_, and RMSD_FL_.

Data-processing and statistical analysis were performed using custom written MATLAB (The MathWorks, Inc., Natick, MA, USA) scripts. Significance of statistical tests was set at α=0.05.

## Results

Ten post-stroke patients were enrolled, equally divided into right and left hemiparetics. Accordingly, ten ND subjects were recruited to serve as gender, age, height and weight matched control group. Table 1 summarizes participants’ features.

**Table 1:** Subjects related features

	**HM**	**ND**
N° subjects	10	10
Gender [F/M]	4/6	4/6
Age [years]	63.0 ± 9.4	59.4 ± 6.6
Range [years]	49-74	50 - 71
Weight [kg]	74.0 ± 9.9	76.2 ± 13.5
Height [cm]	169.0 ± 6.1	171.3 ± 8.4
Stroke onset (months)	8.7 ± 0.6	--
FMA LE (max score, 34)	29.2 ± 1.3	--

Participants’ features. Reported values are mean ± standard deviation.

### Spatio-temporal parameters

**Figure 1:**
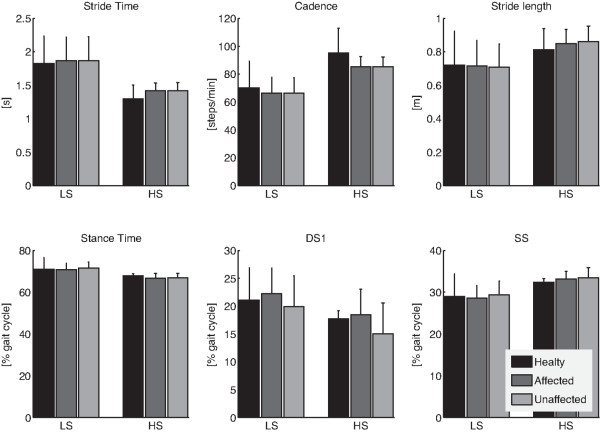
**Spatio-temporal parameters.** Mean and one standard deviation (errorbar) of ST parameters related to healthy subjects (black bars), and hemiparetic patients, both affected (dark gray bars) and unaffected (light gray bars) sides, while walking at low (LS) and high (HS) speeds.

The two-way ANOVA of data related to the HM group (Figure 1 and Table 2) revealed that STs did not differ between affected and unaffected sides but were significantly (p<0.05) influenced by the walking speed. Specifically, cadence and stride length increased in conjunction with reduced stride time and stance time, as result of the increased speed. Regarding stance duration, its timing was modified due to the increased speed involving decreased DS1 and increased SS (Figure 1). No statistically significant interactions between coupled factors were observed.

**Table 2:** Significance of the two-way ANOVA while analyzing STs parameters related to the HM group

**p-values**	**Stride time**	**Cadence**	**Stride length**	**Stance time**	**DS1**	**SS**
Side (affected vs unaffected)	0.948	0.945	0.878	0.514	0.119	0.517
Speed (LS vs HS)	**<0.001**	**<0.001**	**<0.001**	**<0.001**	**0.022**	**<0.001**
Side x Speed	0.550	0.122	0.490	0.620	0.815	0.561

The table reports all p-values obtained by the two-way ANOVA of Speed (HS vs. LS) and Side (affected vs. unaffected) on ST parameters related to the HM group. P-values<0.05 are highlighted in bold.

The comparison between hemiparetic and healthy participants highlighted that the speed was the only factor capable of modifying STs (Figure 1, Tables 3 and 4). In particular, when STs related to both the affected and unaffected sides of HM subjects were compared to those of healthy subjects, after accounting for the variability due to speed, a distinction between the groups was not statistically possible (Tables 3 and 4). No statistically significant interactions between coupled factors were observed.

**Table 3:** Significance of the two-way ANOVA performed on STs parameters related to ND and HM/affected side

**p-values**	**Stride time**	**Cadence**	**Stride length**	**Stance time**	**DS1**	**SS**
Group (ND vs HM/affected side)	0.496	0.302	0.800	0.555	0.590	0.844
Speed (LS vs HS)	**<0.001**	**<0.001**	**0.001**	**0.001**	**0.002**	**<0.001**
Group x Speed	0.550	0.122	0.490	0.620	0.815	0.561

The table reports all p-values obtained by the two-way ANOVA of Speed (HS vs. LS) and Group (ND vs. HM/affected side) on ST parameters. P-values<0.05 are highlighted in bold.

**Table 4:** Significance of the two-way ANOVA performed on STs parameters related to ND and HM/unaffected side

**p-values**	**Stride time**	**Cadence**	**Stride length**	**Stance time**	**DS1**	**SS**
Group(ND vs HM unaffected side)	0.479	0.294	0.759	0.857	0.334	0.545
Speed(LS vs HS)	**<0.001**	**<0.001**	**0.001**	**0.001**	**0.002**	**<0.001**
Group x Speed	0.567	0.132	0.286	0.495	0.477	0.705

The table reports all p-values obtained by the two-way ANOVA of Speed (HS vs. LS) and Group (ND vs. HM/unaffected side) on ST parameters. P-values<0.05 are highlighted in bold.

### Continuous relative phase

**Figure 2:**
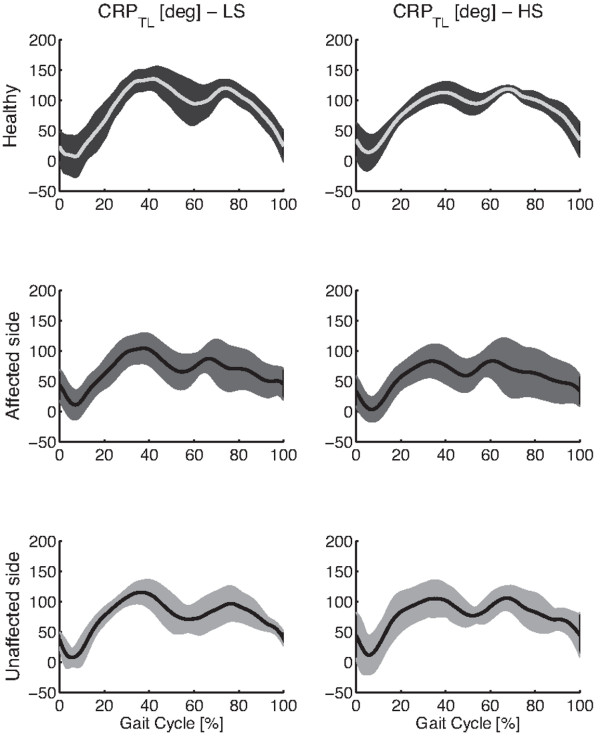
**Thigh-Leg coordination patterns.** The figure shows averaged Continuous Relative Phase (CRP) pattern of the thigh-leg (T-L) coupling, and related one standard deviation wide bands, for healthy subjects and hemiparetic patients, both sides, while walking at LS and HS.

**Figure 3:**
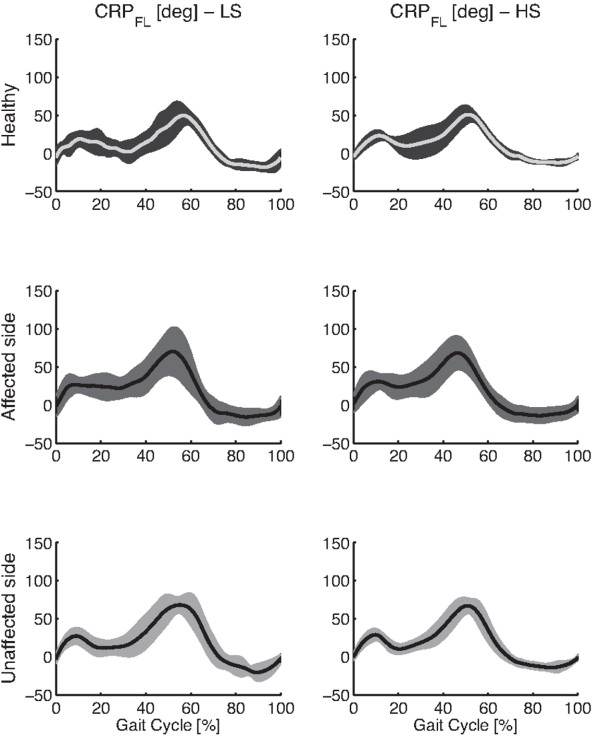
**Foot-Leg coordination patterns.** The figure shows averaged Continuous Relative Phase (CRP) pattern of the foot-leg (F-L) coupling, and related one standard deviation wide bands, for healthy subjects and hemiparetic patients, both sides, while walking at LS and HS.

**Figure 4:**
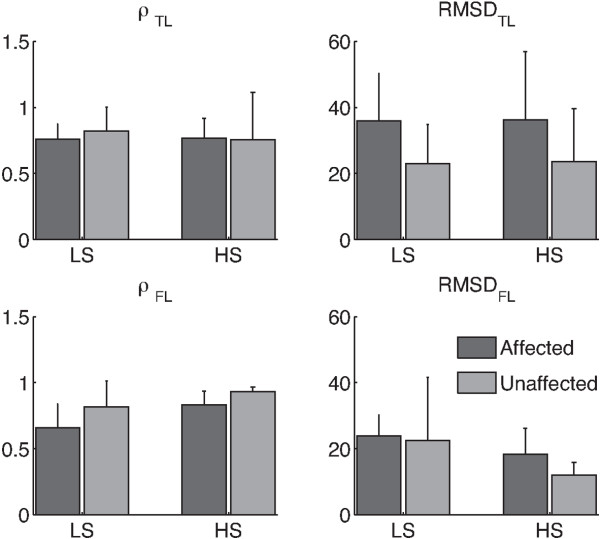
**Metrics describing the CRP.** Mean and one standard deviation (errorbar) of parameters describing the CPR related to hemiparetic patients (i.e., ρ_TL_, RMSD_TL_, ρ_FL_, and RMSD_FL_), both affected (dark gray bars) and unaffected (light gray bars) sides, while walking at low (LS) and high (HS) speeds.

Figures 2 and 3 respectively show the thigh-leg and the foot-leg coordination patterns for healthy individuals and post-stroke survivors, both affected and unaffected legs. Figure 4 reports mean and standard deviation of ρ_TL_, RMSD_TL_, ρ_FL_, and RMSD_FL_.

The two-way ANOVA showed that speed significantly (p<0.05) modified both shape and amplitude of intralimb coordination patterns related to only the foot-leg coupling (Figures 3, 4, and Table 5). In particular, the foot-leg coordination patterns of HM subjects became more similar to those of NDs (i.e., ρ increased and RMSD decreased) as the speed increased (Figure 4).

**Table 5:** **Significance of the two-way ANOVA performed on ρ**_**TL**_**, RMSD**_**TL**_**, ρ**_**FL**_**, and RMSD**_**FL**_

**p-values**	**RMSD**_**TL**_	**RMSD**_**FL**_	**ρ**_**TL**_	**ρ**_**FL**_
Side (affected vs unaffected)	**0.006**	0.263	0.705	**0.005**
Speed (LS vs HS)	0.931	**0.023**	0.662	**0.002**
Group x Speed	0.979	0.460	0.601	0.541

The table reports all p-values obtained by the two-way ANOVA of Speed (HS vs. LS) and Side (affected vs. unaffected) on ρ_TL_, RMSD_TL_, ρ_FL_, and RMSD_FL_. P-values<0.05 are highlighted in bold.

The statistical analysis also revealed significant difference (p<0.05) between affected and unaffected sides in both intra-limb coordination patterns (Table 5). Specifically, the amplitude of the CRP_TL_ and the temporal evolution of the CRP_FL_ of the unaffected side were more similar to that of healthy subjects (i.e., respectively lower RMSD_TL_ and higher ρ_FL_) than those related to the contralateral one (Figure 4 and Table 5).

## Discussion

This study aimed at investigating the attitude of both spatio-temporal parameters and intralimb coordination patterns to capture some neuromuscular adaptations of the hemiparetic locomotion, while comparing healthy and post-stroke subjects walking at fixed speeds on a treadmill. It is important to remark that patients have been explicitly enrolled to constitute a homogeneous group of participants in term of level of motor functioning, age and anthropometric features (Table 1), in order to avoid bias due to the different degree of impairment among them.

### ST parameters and CRP while describing hemiparetic locomotion

As main result, the analysis of ST parameters (Figure 1, Tables 2, 3 and 4) revealed that when subjects walked at matched speeds, their gaits did not show significant discrepancies between the ND and the HM groups, in good accordance with previous literature [4]. In addition, although STs related to the HM group were not characterized by any significant difference between affected and unaffected sides (Figure 1, Tables 2, 3 and 4), the intra-limb coordination patterns of both distal and proximal districts appeared to reflect an asymmetric control strategy (Figure 4 and Table 5). Noticeably, both healthy and hemiparetic participants did not hold any handrail while walking on the treadmill, such that recorded data were not affected by potential upper-body related artifacts [6].

Actually, literature reported contrasting results concerning the symmetry/asymmetry of ST parameters of hemiparetic locomotion. Specifically, there is a wide agreement on the evidence that the asymmetry of ST parameters basically consists of both longer double and single support of the unaffected leg [3] and it has been usually observed when hemiparetic patients walked at self-selected pace [6,26,27]. In spite of this, some authors noticed that the asymmetry can affect different parameters or can be modified by the walking speed. For instance, Chen and colleagues [4] observed that the hemiparetic gait was characterized by a significant degree of asymmetry between legs both in swing time and in step length. Nonetheless, when ST parameters related to both paretic and non–paretic limbs were compared to those of the non-disabled control group at matched speeds, discrepancies between sides and groups disappeared or were characterized by a weaker statistical confidence. Other authors [28] noticed that hemiparetics tended to prolong the swing phase of the paretic limb but did not observe consistent behavior of the step length. Finally, Lamontagne and colleagues [10], in accordance with other authors [3,4,28], stated that the absence of agreement of the direction of stroke-related asymmetries among the studies can probably be ascribed to methodological aspects (e.g., fixed or self-selected pace, numerousness of enrolled groups) and/or to the great variability of the functional level of post-stroke survivors.

Compared to previous studies, in this work, all HM subjects were asked to seek a dynamical equilibrium for given, subject-independent, speeds. They hence adopted suitable biomechanical adaptations leading to symmetric gait cycle timing, but continued to show pathology-related asymmetry only at the interlimb coordination level.

As matter of the fact, the walking speed is a control parameter that affects the metabolic cost of both healthy and hemiparetic subjects [29,30], modifies the dynamic stability of locomotion [31], influences the symmetry between legs in hemiparetic subjects [17,29], and correlates to many ST parameters of hemiplegic locomotion [15]. Regarding presented results, the fixed walking speed, unlike self-selected one, confirmed and extended findings of previous authors [4], revealing that some of the deviations between HM- and ND-related ST parameters can be mainly ascribed to the biomechanical adaptation of post-stroke locomotion rather than to degree of impairment itself. In this regard, the controlled speed can elicit strategies aimed at exploiting the asymmetry of the intralimb coordination patterns without modifying the functional outcomes of the locomotion reflected in the ST parameters. On the whole, these findings support the hypothesis that when the speed is scaled, it probably constraints the impaired Central Nervous System to seek a new dynamic equilibrium that is obtained by separately modulating the coordinative relationship of both proximal and distal segment couplings.

According to this hypothesis, the hemiparetic gait can be achieved by adopting a wide range of possible coordination patterns underlying specific optimization roles, as also observed for neuromuscular injured individuals [32]. Therefore, a constraining locomotion led by a fixed speed, or by an auditory rhythm [5], can hence enable people to gain a significant degree of symmetry, in terms of ST parameters, confirming that suitable cues can improve motor performance. In spite of this, reported results suggest that signs of the brain injury will nonetheless be reflected in other features of locomotion biomechanics such as the coordination patterns. To this regard, the CRP appeared a suitable metric to highlight some pathology-related modifications of locomotion biomechanics. Specifically, since stroke-related geometrical configurations of lower limb segments are compatible with the end-point position observed in healthy subjects, the analysis of the intralimb coordination is expected to better reflect the neuromuscular adaptations describing the rate change of biomechanics resulting after a cerebrovascular accident.

As further result, the analysis of metrics describing the CRP (Figure 4 and Table 5) showed that the thigh-leg coordination pattern was the only hemiparetic gait feature that was not modified by the speed. This behavior was presumably due to the concomitance of two factors: the small difference between LS and HS and the different inertia between proximal leg segments and distal ones. According to these factors, the effect of the speed on the intralimb coordination patterns mainly affected the distal coupling (i.e., Foot-Leg) because it appeared more prone to re-adapt its kinematics in accordance with the imposed speed. However, further experimental tests, in a wider range of speed, are required to confirm this hypothesis.

### Limits of the study

The main limit of this study consisted in the small number of enrolled subjects (i.e., ten per group) resulting in the limited strength of the statistical findings. However, it is worth noted that this limit was needed to reduce, as much as possible, the inherent variability across hemiparetic patients related to the severity of the impairment, the age and the anthropometry. In this respect, the significance of all statistical tests (Tables 2, 3, 4 and 5) was always far from the threshold (i.e., p=0.05) such that results can be confidently considered robust.

## Conclusion

This study demonstrated that when healthy and homogeneous hemiparetic subjects are compared at same speed conditions, the degree of discrepancy between the groups, mainly related to the asymmetry of ST parameters, is significantly reduced. On the other hand, biomechanical adaptations due to the pathology, can be better highlighted in the coordinative patterns of coupled segments within each limb.

These results suggest that the CRP can be more sensitive to the effects of the pathology on the locomotion than the ST parameters. Accordingly, this insight can provide a better understanding of coordinative dysfunctions as a result of pathology and can lead physiotherapists to adopt more suitable treatment interventions.

## Competing interest

The authors declare that they have no competing interests.

## Authors' contribution

The authors equally contributed to this study. Both authors read and approved the final manuscript.
